# A roadmap for sustainable implementation of vocational rehabilitation for people with mental disorders and its outcomes: a qualitative evaluation

**DOI:** 10.1186/s13033-023-00620-8

**Published:** 2024-02-10

**Authors:** Yvonne Noteboom, Femke van Nassau, Astrid R. Bosma, Eric J. E. van der Hijden, Maaike A. Huysmans, Johannes R. Anema

**Affiliations:** 1 Amsterdam UMC, Department of Public and Occupational Health, Room C379, Van Der Boechorststraat 7, 1081 BT Amsterdam, The Netherlands; 2 Faculteit Der Sociale Wetenschappen, Talma Institute Vrije Universiteit Amsterdam, De Boelelaan 1105, 1081 HV Amsterdam, The Netherlands

**Keywords:** Work participation, Mental health care, Social security sector, Mental disorders, Stakeholder collaboration, Funding, Implementation, Vocational rehabilitation, Financial implementation strategy, Shared savings strategy

## Abstract

**Background:**

People suffering from mental health disorders have lower work participation compared to people without mental challenges. To increase work participation within this group vocational rehabilitation interventions are often offered. Collaboration between the mental health care and social security sectors is needed to enable professionals to perform optimally when carrying out these interventions. Yet, regulatory and financial barriers often hinder sustainable implementation. To overcome these barriers an experimental roadmap for sustainable funding based on a shared savings strategy was piloted in four regions. The aim of the present qualitative study was to gain understanding of the uses of this roadmap and the factors that were important in the experiment’s process.

**Method:**

The roadmap consisted of five steps based upon insights from shared savings strategies and implementation science knowledge, and was initiated by a national steering board. The roadmap aimed to make sustainable funding agreements (based on shared savings) for the implementation of a vocational rehabilitation intervention. In four regions, stakeholders from the mental health care and social security services sector followed the roadmap. We conducted interviews (n = 16) with involved participants and project leaders of the experiment and collected 54 sets of field notes and documents to evaluate the roadmap process. A thematic analysis was used to analyse the data.

**Results:**

Regions perceived improved stakeholder collaboration around vocational rehabilitation after they were guided by the roadmap. Three regions made, or intended to make, agreements on collaboration and funding, yet not based on shared savings. Moreover, going through the roadmap took more time than anticipated. Stakeholder collaboration depended on factors like personal and organizational interests and collaboration conditions and values. Financial legislation and politics were regarded as barriers and personal motives were mentioned as a facilitator in this process.

**Conclusions:**

Our study showed that the roadmap supported stakeholders to establish a more sustainable collaboration, even though no sustainable financial agreements were made yet. Although participants acknowledged the function of financial insights and the need for financial resources, the driver for collaboration was found to be more on improving clients’ perspectives than on solving unfair financial distribution issues. This suggests modifying the focus of the roadmap from financial benefits to improving clients’ perspectives.

**Supplementary Information:**

The online version contains supplementary material available at 10.1186/s13033-023-00620-8.

## Introduction

Unemployment rates are higher among people with mild and severe mental disorders than among the general working population [1, 2]. Unemployment results in a higher use of social benefits, lower life satisfaction, less social participation and a higher risk of poverty [1]. The populations that receive social benefits and mental health care overlap to a large extent: one third of people receiving social benefits also receive mental health care and 60% of governmental mental health care costs are made by unemployed people [1]. At the same time, studies have shown that work can have a positive influence, not only on individual health, social interactions and income but also on costs for society [1–3]. If work participation among people with mental health problems increases, the use of social benefits and health care declines. Both, the social security sector and mental health care sector can eventually benefit from increased work participation in this group [1].

Studies have shown that many people with a mental disorder have a desire to work, but various factors impede them from successfully entering paid employment, such as (self-)stigma and fear of failure [1, 4–6]. Financial and legislation barriers, like the risk of losing benefits or not being eligible for inclusion in a vocational rehabilitation program are also mentioned as inhibitors in the literature [1, 7].

Many vocational rehabilitation interventions have been developed to support people with mental disorders to overcome these barriers and increase work participation [8, 9]. There are many forms of vocational rehabilitation interventions, mostly focussing on (job) skills training, job search support and other employment services [8, 9]. Several of these interventions are found to be (cost-)effective [8, 10–13]. These interventions often ask for an integration of mental health care and social security services, as clients need support from both fields (e.g. during supported employment, a frequently cited, evidence-based example is individual placement and support (IPS)) [1, 8, 14–16]. Indeed, vocational rehabilitation interventions where these two services are integrated are found to be more effective [1, 8, 17, 18].

Despite the fact that integration of mental health and social security services are associated with improved employment outcomes, sustainable implementation of vocational rehabilitation is lacking due to insufficient collaboration, regulatory barriers and insufficient funding [1, 14, 16, 19–22]. Frequently mentioned barriers are: policy and laws not being in line with intervention goals, continuous changing legislation and fragmented and temporarily funding [19–21, 23–29]. While various vocational rehabilitation interventions have been implemented around the world over the last decade, a number of interventions also ceased to exist [19, 26].

In the Netherlands, a national steering board aimed at increasing work participation for people with mental health problems stated that the lack of sustainable implementation of vocational rehabilitation interventions is caused by short term funding. This implies that collaboration stops when the funding is ceased—a trend, also seen in literature [25, 30]. In the Netherlands, IPS is so far the only intervention that has recently been structurally funded, yet even this is only for a portion of potential clients [31]. Costs of vocational rehabilitation are made before and during the application of the intervention. While savings occur after this, when employment is reached. Government solutions for this are often presented in temporarily subsidy schemes [21, 30]. The Dutch steering board hypothesized that embedding a sustainable funding model based on a shared savings strategy for the application of vocational rehabilitation interventions could lead to improved and more sustainable integration of care between professionals in health care and the social security sector. Therefore, optimizing vocational rehabilitation services for people with mental health problems. As far as we know, no scientific articles have reported on the use of a shared savings strategy in the field of vocational rehabilitation for people with mental health problems. But the strategy has found to be potentially useful in therapeutic healthcare settings [32–34]. In the therapeutic health care, implementation of shared saving agreements have been shown to be a successful strategy resulting in positive outcomes for the continuum and quality of care, as well as expenditure savings [32–35].

To test this hypothesis, the steering board initiated an experiment (“Meedoen, Meedelen”: “To share, to Participate”) wherein a roadmap was designed aiming to make shared savings agreements between the mental health care and social security sectors to stimulate sustainable collaboration around vocational rehabilitation for people with mental health problems [36]. The aim of the present study was to gain understanding of the uses of this roadmap and the factors that were important in the experiment’s process.

## Methods

Between January 2020 and May 2022, a qualitative study was conducted to gain understanding of the implementation of the roadmap for sustainable funding in four regions in the Netherlands. Data collection consisted of semi-structured interviews, field notes and review of project documents.

### Setting

In the Netherlands, mental health care and social security services are provided in different financial and regulatory sectors [37–39]. Most of the time, mental health care providers are responsible for implementing vocational rehabilitation programs that aim to increase work participation of people with mental health problems. Stakeholders in the social security and services sector have several roles and tasks when it comes to work participation, including paying out unemployment benefits and refer to and financing vocational rehabilitation interventions. They are also responsible for other relevant care and support, like adult support services, social support and job coaching. Stakeholder responsibilities and tasks can depend on national and local policies. Detailed information on the Dutch context of the legislation and financial organization of mental health care and vocational rehabilitation can be found in Additional File 1**.** To improve collaboration across sectors between stakeholders, a Dutch national agreement was signed in 2019 aiming at *“effective job-(re)integration for people with a psychological vulnerability or retention of work. So that dropout from the labour market and further social loss (debt, poverty) is prevented, including the improvement of work participation for people with mental disorders”*. This agreement was extended in 2020 [40], and a national steering board (‘Sterk door Werk’; Stronger through Work) with representatives from nationally involved stakeholders was founded which initiated the experiment described below [36].

### The experiment

#### Emerge of the experiment

As mentioned in the previous paragraph, the ‘Stronger through Work’ steering board members concluded that financial and regulatory barriers such as temporarily funding, hamper sustainable collaboration between stakeholders working in the field of vocational rehabilitation. More specifically, the board members believed that when funding stops stakeholders are not able to continue to finance vocational rehabilitation interventions. As a result, the interventions and collaborations cease. The steering board members reasoned that this is caused because the ones that need to invest upfront (mental health care providers) are not the ones that receive the most benefits from this investment (i.e., reduced payment of unemployment benefits/disability pensions) afterwards. It is rather the social security providers and health care insurances providers who receive the greatest reward when employment resumes. Funding can close this gap, but is only temporarily effective. However, the board found that in other health care settings innovative financial agreements are being utilized that can lead to improved integrated care [34, 41, 42]. Based upon these findings, they selected the so called ‘shared savings strategy’ to tackle the lack of sustainable funding and collaboration.

#### Shared savings strategy

With a shared savings strategy health care stakeholders (like providers and insurers) first explore aspects of the current quality of health care for a specific target group [34]. Improvements for quality of care and outcomes for this specific target group are developed and agreed upon by health insurers and health care providers. The costs necessary for the improvements in quality of care (investment) are then calculated. As well as the return-on-investment for these costs (= savings) if the intended outcomes are achieved, under the agreed quality of care conditions [34]. Part of these agreements is that the ones that invest are also the ones that benefit most from the savings, so they can re-invest in outcomes and quality again [34]. In this way, the shared savings strategy can stimulate structural funding and more sustainable collaboration due to shared accountability for costs and quality of care [34].

Seeing an opportunity to learn from these insights, the national steering board members translated this model from the health care sector to the vocational rehabilitation sector and the above mentioned experiment was initiated.

#### The roadmap

A national project group consisting of members from the national steering board supported by a project leader and researchers translated the board’s idea into practice by developing an implementation roadmap based on the shared savings strategy used in health care settings and supplemented by other implementation strategy principles: a local needs assessment, identifying barriers, and the development of an intervention [34, 41, 43]. The roadmap contains five steps. The first step is recruiting relevant stakeholders to participate in the experiment. The second step is to select or develop a specific type of vocational rehabilitation intervention for a selected target group (step 2a) and calculate costs and potential benefits of the vocational rehabilitation intervention (step 2b; business case). The third step is to make a detailed work plan, describing the allocation of activities and costs to implement the selected vocational rehabilitation intervention. Step four is to formalize the collaborative agreements on the detailed work plan, and step five is implementing and monitoring the collaborative agreements. The proposed duration of the experiment was one year for going through the first four steps and the implementation of the intervention and three years for monitoring (step 5). Details of every step are explained in additional file 2.

#### Organizational structure

**Fig. 1:**
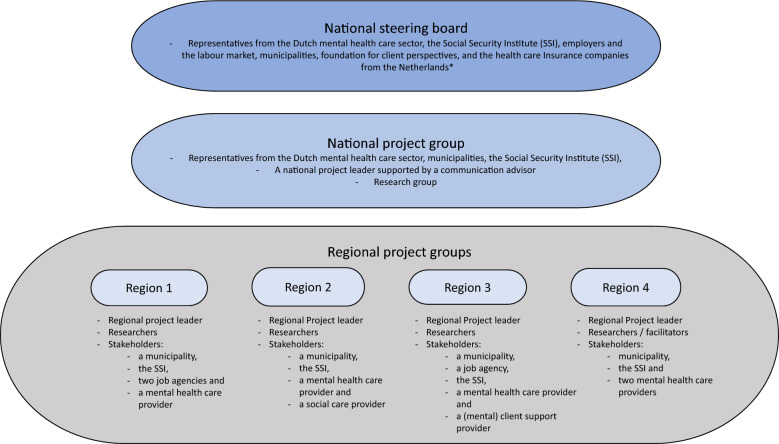
Structure of project organization. * National representative of the Dutch mental health care sector, the Dutch Social Security Institute (SSI), the Association for an inclusive labour market, the Association for municipalities in the social services sector, the Foundation for mental health and well-being, the Foundation against stigma, the Dutch association for municipalities, professional associations for insurance physicians and employment experts, the Employers association and an advocacy organization for health care Insurance companies in the Netherlands

The experiment was funded by the Dutch Ministry of Social Affairs and Employment (SZW) and consisted of a national level project group and four regional project groups. The national project group was comprised of four representatives from the national steering board, complemented with researchers (the authors of this paper). For details on the organizational structure see Fig. 1. The national project group’s responsibilities were to set up a roadmap, to introduce this roadmap in four regions, to facilitate these regions’ use of the roadmap and to evaluate the development process, the implementation process and client outcomes for the selected vocational rehabilitation intervention. This paper is focused on the evaluation of the development process, results of the latter two evaluations will be described elsewhere, as data collection is still ongoing.

In this experiment, the national project leader was responsible for recruiting regions and explaining the experiment (step 1). The national project leader supported the researchers and regional project leaders during all steps: kept them up to date about relevant topics, regulated the progress of the regions and informed each region about the other’s achievements, a learning network was thereby created. The national project leader was also responsible for reporting to the national steering board. The regional project leaders had a facilitating role in collecting regional stakeholder representatives (step 1), preparing regional sessions (step 2), drawing up, presenting, and implementing outcomes (step 2–5), supporting collaborative agreement making (step 4) and the follow up of these agreements. The researchers had a facilitating role in supporting regions during the experiment: they facilitated the regions’ progress by explaining the experiment (step 1) and, they supervised and facilitated multidisciplinary group sessions and one-on-one sessions (step 2a). They also gathered data to develop the business cases with the help of an external econometrist (step 2b) and supported the development of the work plan (step 3). Researchers additionally attended sessions about formalizing and implementing agreements (step 4 and 5). Depending on the stakeholders’ needs per step, multidisciplinary group sessions or one-on-one sessions with individual stakeholders were held. Additional file 2 explains the details of every step of the stepwise approach.

#### Recruitment of regions

Out of 35 existing labour market regions in the Netherlands, four regions were selected for this project using a pragmatic approach aiming for a broad geographical spread and variety in region size. The inclusion “criterium” for regions to participate was: having an interest in improving collaboration between mental health care and social security sectors aiming to improve work participation for people with mental health problems. After eight regions were approached for the experiment four agreed to participate. Although all regions showed interest in the set-up of the experiment four out of eight regions had reasons for nonparticipation: already participation in a comparable project or study, not feeling the need for the experiment (because collaboration between sectors was already considered sufficient) or not having enough time or financial resources. After the regions were finalised and a regional project leader was assigned to each region, implementation of the roadmap began. See Fig. 1 for details of the participating stakeholders in each region.

### Data collection

Several qualitative research methods (triangulation) were used to collect data, including 16 semi-structured interviews, 54 sets of field notes from different meetings and an analysis of documents per region [44]. Data was collected from January 2020 through May 2022. When regions ended their roadmap sessions the experiment was considered to be over. Some final interviews were performed and then data collection was concluded as well.

#### Interviews: interview structure

Semi-structured interviews with participants involved in the experiment were held to gain more in-depth information. In total, 16 interviews were conducted by the researchers (YN, AB). Interviews were held at the beginning, during the middle, and at the end of the experiment to obtain a comprehensive overview. The semi-structured interviews were held in Dutch and – due to COVID-19 – done by video conference. An interview guide with open-ended questions was used to structure the interviews and ensure comparability. Interview topics were related to the stepwise approach of the experiment and to the national and regional contexts, including questions regarding collaboration among regional stakeholders, the stepwise approach and participant’s own roles within the experiment. Interview guideline can be found in Additional file 3. Interview topics iteratively changed over time, depending on the ongoing steps in the stepwise approach of the interviewees. Interview duration ranged from 60–90 min.

#### Interviews: interview participants

Interview participants included, the national project leader, three regional project leaders, four regional stakeholder representatives, three national project group and three steering board members. Participants were sampled purposively, and all had a role within the experiment. Two project leaders were interviewed twice (half-way and at the end). All interview participants signed informed consent. Data collection from interviews was completed when new themes were no longer emerging.

#### Field notes and document analysis

Two researchers (YN, FN) were present during all experimental sessions including multiple and single stakeholder meetings and project leader meetings. Field notes were taken by one of the researchers. If the researchers were not able to take notes during the group sessions, sessions were recorded. Fifty-four sets of field notes were analysed, from which 35 were based on group sessions including multiple stakeholders and 17 from one-on-one sessions with an individual stakeholder. Stakeholder representatives during sessions varied from operational employees, (data-)managers and policy makers to regional directors. All type of stakeholders were spoken to at least once in a one-on-one session. In each regions different professionals function levels were present in the roadmap sessions. Relevant documents like brainstorm outputs, project plans and regional contract documents were collected during or after sessions.

Researchers (YN, FN) were present in all four regions during all steps; this enabled the researchers to collect data at all stages of the stepwise approach. Both researchers YN and FN made field notes during every session they attended and then shared and compared them directly after the sessions, to enrich observations. Relevant documents, such as formalized contractual documents or work instructions, were obtained from the regional project leaders to complete the data.

#### Ethical considerations

The study was assessed by the Medical Ethics Committee (METC) of Amsterdam University Medical Centre – location VU University Medical Centre (2020.0610), which declared the protocol did not fall under the scope of the Medical Research Involving Human Subjects Act (Dutch law). The study was conducted according to the Declaration of Helsinki.

### Data analysis

All interviews were audio recorded and transcribed verbatim by a specialized external agency. Audio recording of group session were transcribed verbatim by YN. A thematic analysis method was used to analyse the data [45, 46]. Coding was conducted with MAXQDA 2020 software. Interviews and field notes were used for thematic analysis, field notes and documents were used to evaluate the roadmap progress.

#### Interviews and field notes

First, three interviews and two sets of field notes were analysed with open coding (by researcher one, YN).Two of these interviews and one set of field notes were also analysed by two other researchers (AB and FN). The codes were then compared and checked to reach consensus. Initially, the codes were organized using the Consolidated Framework for Implementation Research (CFIR) [47] by researchers YN, FN and MH. Researchers found that codes often related to two CFIR domains moreover ideally placed on the border of two CFIR domains, and therefore codes were relabelled thematically. The thematic ordering was discussed by the researchers involved in the thematic analyses (YN, AB, FN & MH) to reach consensus before the rest of the data was analysed. The codebook can be found in Additional file 4. When the lead researcher (YN) was in doubt about the interpretation of the data other researchers (AB, FN, MH) were consulted. Additional sessions with all researchers (with different disciplinary backgrounds) were held to interpret the data and come to a final consensus about the content and ordering of the themes.

Quotations were obtained from both the interviews and field notes and translated from Dutch to English by YN. Translation was discussed with the other researchers. Back translation was not performed.

#### Document analysis

Additional documents were also analysed to obtain information about region characteristics and collaboration structure.

## Results

The four participating regions differed in: in for example collaboration starting point and stakeholder composition and progressed differently through the roadmap steps. Descriptions of starting point characteristics, collaboration needs, and agreements on funding are provided per region in Table 1. The developed interventions are explained in more detail in Box 1. Moreover, details of the roadmap progress per region and contextual aspects can be found in Additional file 2.

**Table 1:** Progress per region: Description of progress—Collaboration, intervention, and agreement development per region

	Region 1	Region 2	Region 3	Region 4
Starting point characteristics (step 1)	Stakeholders from both fields were already working together in a project group on mental health and work participation. Collaboration was perceived as good Gathering relevant representatives from every stakeholder group was organized easily and the project group remained stable Two collaboration projects on vocational rehabilitation based on temporary partnership agreements were running, which regional stakeholders developed prior to the experiment	Stakeholders from both fields were already working together on a pilot level Agreements on the pilot were running and monitored Gathering relevant representatives from every stakeholder group was organized relatively easily and the project group remained stable	Professionals from both fields already knew each other from some previous collaboration initiatives that had run, but no specific collaborative agreements were active at the beginning of the roadmap sessions Still, gathering relevant representatives from most of the stakeholder groups was organized relatively easily and the project group remained stable	Stakeholders from both sectors were working together in this region, though separately by one-on-one agreements Gathering relevant representatives from most of the stakeholder groups was relatively difficult as it was unclear which representatives were needed The experimental project group was not stable and stakeholder representatives were inconsistently present during meetings
Collaboration needs & Developed intervention (step 2–3)	Two interventions were running in a pilot phase: the *Developmental Labour-related day care (LDD)* and *Job oriented integrated treatment approach (JIT)* are found in Box 1 Needs for improvement and sustainability for the in-progress collaborations were mentioned	Three collaboration initiatives were started: First, an individual placement and support (IPS) professional from the health care provider was added to the neighbourhood teams Second, structural interagency meetings to discuss job (re-)integration possibilities for the neighbourhood clients were introduced. Details of these *work-focused interagency meetings* (WIM) are found in Box 1 Third, the implementation of the *job oriented integrated treatment* (JIT) *approa*ch (adopted from in region 1) was proposed Needs for improvement of the in-progress collaboration were mentioned	The project group participants developed a pilot of *work-focussed interagency meetings (WIM)*. Details of this intervention (3) are found in Box 1 Needs for collaboration improvements were indicated by the experimental project group	No specific intervention was developed during the roadmap sessions, though some wishes were expressed Needs for collaboration improvements were related to the extension of in-progress one-on-one agreements
Agreements on collaboration and funding (step 4–5)	Intervention 1 was embedded in local policy by funding based on fee for service and intervention 2 was supported by pilot funding and embedded in organizational structure, after the region went through the roadmap steps	Adding the IPS professional to the multidisciplinary teams was formalized by the mental health care provider. By the end of the roadmap sessions, a regional steering board gave a positive recommendation for implementing the two interventions (2 and 3) but no decision was made yet	Stakeholders started with their developed intervention on a small-scale base, the municipality decided to support this with small-scale pilot funding (need-based payment) and organizational assistance, based on the alderman’s decision	By the end of the roadmap session no specific collaboration or funding agreements were made by the stakeholders but the intention to set up a broader regional collaboration initiative was expressed

Box 1. Details of the developed intervention in the regions * In the Netherlands the term day care can also refer to “dagbesteding”, this can be described as an "adult support service" where people go to organized daily activities while they are supported on different aspects (like physical or mental support).** in region 2 clients were included in this intervention after pro-active caseload screening *** in region 3 clients were included in this intervention when clients were identified as so called “stuck cases”, which means that regular care and support was not suitable for these specific clients.

1. Labour-related developmental day care (LDD) (region 1): this is a specific job participation oriented adult support service. Clients within day care services* are regularly not employed. Clients who want to work are applicable for this specific day care placed in a working environment, located at job reintegration agencies. Mental health care and job reintegration professionals are working together in the support of clients. Moreover, the municipality is involved by facilitating access to and financing the labour-related developmental day care.
2. Job-focused integrated treatment approach (JIT) (region 1 and 2): this intervention is an integrated approach of treatment and job reintegration. A specific work-focused cognitive behavioural therapy (w-CGT) provided by the mental health care provider is integrated with a job search supported by the SSI and the job reintegration agency. Professionals from the Mental health care, SSI and job reintegration are working together in the support of the clients. Agreements exist of embedding this intervention within the regular care by all professionals. First this intervention was done ‘pro deo’ on the personal interest of professionals, after a while the SSI supported some pilot funding, making some capacity available.
3. Work-focused interagency meetings (WIM) (region 2 and 3): this intervention is an integrated approach where professionals from multiple stakeholders come together on a regularly base to discuss about work participation possibilities for one or more clients (**/***). Steps of the interagency meetings were described during roadmap sessions:
  - Discuss on possibilities professionals see on clients wishes on work participation.
  - Obtain Clients’ need on support and possibilities to fill in those needs.
  - Decide which professional & organization is the most suitable to provide the needed support.
  - Making agreements about the coordination, application and, goals and feedback of the chosen support.

### Thematic findings

After analysis of the semi-structured interviews and field notes, four themes with ten subthemes on factors influencing the roadmap progress were identified in the data.

### Motives and interests

Several motives and interests for participating in the experiment were mentioned by participants related to their willingness to participate and their process in following the roadmap sessions, including both personal motives and organizational interests.

#### Personal motives

Personal motives were mentioned by participants regardless of their functioning level and type of organization. A common driver to participate in the experiment reported to be: improving the quality of care and life for clients. Societal gains were also mentioned to be an important factor.

‘ All in interest of the client’ – Manager SSI.

Moreover, participants mentioned that client goals were more important than financial gains. In addition to this, participants mentioned that they were in search of proof for their gut feeling that their personal investment in and support of clients in vocational rehabilitation would lead to improved client well-being.

*‘But I want to emphasize again: first I was also about quantities and the financial aspects. But now, first we look at what we want to achieve [for the client], then we *look* how, financially. This is also important for the story.’ – Director of a job agency.*

#### Organizational interests

In terms of organizational interests, reducing the number of clients receiving social benefits were mentioned by participants from municipalities and the SSI. Mental health care professionals discussed wanting to serve more clients. Financial incentives, such as decreasing costs and obtaining funding, were only mentioned by project leaders and participants from municipalities and health care insurances.

*‘Still, we are a money-driven organization so we hope to achieve structural financing through this project / research.’ –* Municipality manager.

Other organizational interests that were mentioned included: improving expertise, broadening organizational scope, and having an interest in short-term and long-term financial and client outcomes. Organizational interests were also related to societal gains, e.g., reducing the number of people in the benefits was understood to reduce crime. On the other hand, mostly field professionals mentioned that they had the feeling that their personal and client goals did not always meet organizational goals, for example when improving the societal participation of clients does not always lead to a decrease of the use of benefits but can improve client quality of life.

*‘There are societal benefits also in it. Sometimes you can help somebody. From nothing to something, like helping in a nursing home for two hours a week. I also think that’s a profit. There are no savings on the benefit payments, so sometimes you receive internal criticism. But you want help people move forward’.* – Professional SSI.

### Collaboration

Collaboration was a key element in this experiment. Important factors that were found to impact collaboration included the conditions and values under which people chose to participate, their reasons for participating, and the structure and organization of the entities of which they were a part.

#### Conditions and values

Participants mentioned that knowing each other, aiming for the same goal, understanding each other’s perspectives and having trust in each other were all characteristics of a well-functioning collaboration.

‘*There was already a reasonable base to talk with each other; people know each other, are aiming the same goal and have sympathy for each other’s perspectives’*. – Mental health care professional.

*‘Well yes, when I need somebody, I call that one. You know, so that makes…., because you know each other and understand each other perspectives, …it easier to call each other.’*—Mental health care professional.

Setting up a collaboration takes both time and commitment. In two regions a prior history of a collaboration was seen as a distinct advantage. During sessions, participants showed more mutual knowledge and shorter lines of communication if their relationship pre-dated the experiment. Participants pointed out key elements of collaboration which were: functioning on the same level, structured organization and specific key persons that initiate and consolidate collaboration.. However, a lack of continuity makes collaboration more difficult, as illustrated by the quote below.

*‘But yes, then [project leader x] left, there was no owner, no driving force anymore and then it becomes difficult.’ –* Mental health care professional.

#### Reasons for participating in collaboration

Reasons for collaboration between stakeholders raised during sessions included: sharing expertise and knowledge and, improving organizational alignment and cliental support. Reasons mentioned for not wanting to collaborate were disinterested in looking beyond their own organizational interests and, having the feeling that the collaboration had no added value because it was less efficient or not useful. Participants also showed different perceptions on client goals and perspectives making collaboration more complex.

*‘Not relevant to us. We only look at our municipality, not at the SSI’ –* Policy maker municipality.

Other reasons that kept participants from collaborating were not wanting to work in favour of other organizations’ benefits and the feeling that they were in competition of the same client goals and outcomes, e.g. in searching for jobs and employers.

*“But still, we are fishing in the same pond, for vacancies and employers”* – Social security professional.

#### Collaboration structure and organization

Due to legislation, stakeholders from different sectors have different client goals (like curing vs. helping them obtaining employment) and, different tasks (like paying benefits or caregiving). Moreover, stakeholders differ in size and geographical demarcations between client groups that stakeholders serve were observed, making collaboration difficult. Goals, tasks, and working areas overlapped partly between stakeholders but never completely. These structures are historically grown, based on legislation, and ingrained in the way professionals work. This complicated determining a common target group, among other things.

*“[Stakeholder X] is not involved [in this collaboration] because the postal area of clients they work with differs. In [city X] there’s a good overlap of the postal areas they work with so there they are involved in the collaboration.”* – Regional project leader.

Financial and collaboration structures between stakeholders changed over time due to regional policy changes; for example, when the services of one of the stakeholders was not purchased by another stakeholder anymore. Also, several similar collaboration plans initiatives were initiated during and interfered with the experiment (e.g., a nationwide parallel funding initiative aiming to increase the number of supported employment interventions). For participants these changes were seen both to be a complication (making the collaboration vaguer and more insecure) and a chance (good timing for introducing new initiatives or collaborators).

‘Yes, something changed in this city. This [day care*] provider is not covered by the collaboration structure from the municipality anymore. So from that point on, [day care] from this [day care] provider could only be used for the first three months of care and then the professional need to look for another day care provider’ – Social care professional.

* adults *support* service, see also explanation Box 1.

### Politics, finances and legislation

Politics, finances and legislation played a role on different levels through the experiment, including political trade-off and financial political interaction. Moreover legislation was hindering participants on different levels and organization of funding was found as a theme.

#### Political trade-off and financial political interactions

Political trade-off played a role in collaboration between stakeholders and were evident during the experiment. Participants showed some reservations regarding the financial aspects: sentiments around unfair finance and task division played a role in this and hindered trust among stakeholders, while this was the actual reason for starting the experiment. At first, distrust kept participants from investing money, especially when the application of the intervention or the profit lay with another stakeholder.

*’I do all the work and mental health care gets funded. That doesn’t feel good.’* – Professional SSI.

Support from regional political leaders was mentioned by participants and project leaders as being very important in the continuation of their collaboration, especially in terms of obtaining funding. But it was difficult to determine at what point these political leaders should be involved: interests and political hassle were pointed out as important factors in this.

*‘Everything is with reservation/caveat of the opinion of the Alderman for the municipality/ municipality city councilman’*. – Mental health care professional.

*“Something is going on within a related collaboration pilot. It has something to do with the decision making by the alderman for the municipality, it’s complicated… there is a lot of talking and ‘moulding’ going on, to plan the needed meetings. I have some concerns about what’s needed and the relevance for the continuation of this experiment*’. – Regional project leader.

Moreover, participants mentioned political interests for the outcome of the experiment: political leaders need a supportive narrative to defend decision making and their political and financial responsibility. A business case and showing the proof of (cost-)effectiveness were mentioned as supportive tools for both obtaining support from decision makers, as these provide insights and substantiation.

*‘Our local alderman is well informed, is interested in this project but needs a proper narrative for the city council to say: “we should continue this”.*’ – Policy maker municipality.

Some participants even expressed that business case outcomes are essential for convincing decision makers like managers, directors, and political leaders. On the other hand it was also mentioned that if a political leader wants to achieve something money is no issue, especially when political leaders show an affinity with the target group.

*‘How we get money for this is still a question… this is difficult. But yes, when our alderman wants something, either left or right, then it will be achieved.’* – Policy maker municipality.

#### Legislation barriers

Participants felt dependent on both local and national legislation and existing funding systems. This is institutionalized in (organizational) system thinking and IT features. In practical terms, professionals were not allowed to access certain administrative or IT systems. This made data needed for the business case calculations inaccessible. Sharing information (like on the use of benefits and care) between stakeholders was also prohibited and participants did not feel they had capacity to take on resolving this barrier. Finally, involved professionals showed frustration at not being allowed to use certain vocational rehabilitation interventions with their clients because they had not been purchased by their organization or they do not receive a reimbursement for it. Participants expressed they wanted to resolve this. These barriers made determining a common target group and setting up a business case time consuming and complex.

*‘I see a lot of obstacles within the organization, a little sneak peek: “This is an tightly organized organization. This is allowed and this is not*’. – professional SSI.

Legislation changed during the experiment, which made agreements between stakeholders prone to instability. The legislative changes sometimes counteracted existing collaborative goals or resulted in new parallel goals being drawn up (e.g., an introduced subsidy scheme was only made available for one type of vocational rehabilitation intervention (IPS)), which was not in line with some collaboration agreements stakeholders were planning to make.

#### Organization of funding

Participants mentioned legislation prohibited them from finding financial means to invest in advance—which was needed to make the intended shared savings agreements. For example, the legal ground of financial resources was hindering. Other financial barriers mentioned included lack of financial resources to invest and the bureaucratic process for obtaining funding.

‘*Yes, you know, they want me to also pay for it but I can’t pay because I’m out of financial resources and I don’t have lawful grounds to pay*’.—Health care insurance professional.

Still, existing barriers were bypassed by two of the four regions. These regions did made agreements on funding for the investment of the developed pilots (Box 1). Participants in these regions made no shared savings agreements but suited agreements to the regional situation: they did agree on fees for activities by a subsidy scheme, based on stakeholder intention to invest in the target group.

### Complexity of a new experiment

The concept of ‘shared savings agreements’ was rather new for the involved professionals of both sectors and this novelty has influenced the process according two different aspects: the conceptual and the organizational aspect.

#### Conceptual aspect: goal and concept of the experiment

At the beginning, participants mentioned that the overall goal of the experiment was not clear to them. During sessions they discussed finances, collaboration, and client outcome goals. Moreover, participants discussed about which aspect was more important and whether these can co-exist, before reaching consensus. For participants, the concept of ‘breaking down barriers’ to integrate care among two sectors was experienced as conflicting because they had the feeling that in fact some boundaries needed to be set to concretize collaboration agreements.

*‘On one side I think, let’s *just* sit down and decide but on the other side we need to have some boundaries.’-* Mental health care professional.

The idea of investing first before receiving savings was clear for participants but the arrangement of this felt difficult, especially with regard to providing money on advance. Likewise, in the development of the business case, participants experienced complicating factors, such as deciding which costs were supposed to be relevant and the difficulty of predicting costs, outcomes and benefits for a newly developed collaborative intervention. To overcome this, the business case had to be discussed in several meetings with professionals, policy makers, and directors to reach consensus. Making scenarios, supported by business case calculations, was experienced helpful in reaching consensus.

#### Organizational aspects

The experiment consisted of different steps on different topics, like determining the target group and realizing funding, which made the organization of the experiment complex for participants. Moreover, it was unclear which stakeholder representative was needed during which step, so ‘missing’ or ‘wrong’ stakeholder representatives were seen during multiple sessions. This led to a repetition of comparable sessions, which took time. Subsequently, new participants were needed for the next step, so the experiment had to be explained multiple times.

Participants mentioned that they had no clear picture of the required preconditions, which hindered decisive action (i.e., regarding inclusion and exclusion criteria for the target group, the continuation of the experiment, and maintaining of the savings). The set-up of the roadmap and the facilitating support by the project leaders and researchers during the sessions by was experienced helpful in going through the roadmap, making the experiment and the needed activities more clear.

*‘Where are we now in the project? I do understand the relevance. There’s a project plan but I feel like I don’t really understand how we get here. Now we are looking back at the execution of supported employment and how it’s *going*, I don’t really understand. First, we had the health care insurance company involved as well and also other parties. Where are those parties?’* – Mental health care manager.

The volume of the experiment was felt to be too big, participants wanted to start with a smaller pilot in terms of numbers or for a shorter amount of time. Starting the collaboration on a pilot level with a small number of clients was experienced successful as a way to implement the intervention.

’Hundred *clients is *ambitious*. Why not start with ten, learn from it and if results are positive expand from there?’*—Mental health care manager.

Finally, participants indicated that decision makers can be supported by smaller decision point, intermediate and at the end. Mostly, these decisions points were introduced between steps 2a, 2b and 3 (e.g., verifying the target group, the goal of collaboration and the business case outcomes).

## Discussion

### Main findings

In this qualitative study we investigated the factors that were important in implementing a roadmap, based on a shared savings strategy, that aimed to induce sustainable funding for vocational rehabilitation services in the mental health care and social security sectors. For this we followed four regions that implemented the roadmap. Participants experienced improved stakeholder collaboration in vocational rehabilitation services after they were guided by the roadmap. This can be substantiated by results of new or renewed agreements on collaboration and funding that were made between stakeholders from the mental health care sector and social security sector or the intention make such agreements. Yet, not all regions were successful in making agreements and making such agreements took more time than anticipated. The of participants’ personal motives for improving the client quality of life and existing collaboration among stakeholders (i.e., already knowing each other and having the same goal) were found to be important facilitators. On the other hand, legislation and having different organizational structures and interests were barriers in this collaboration process, hindering trust, sharing information, and finding a common target group. Politics, finances, and changes in collaboration structures were found to be important factors that could work either positively or negatively in the process. Participants acknowledged the function of financial insights and the need for financial resources to implement a collaborative vocational rehabilitation intervention but financial incentives were not found on an individual level.

### Key elements in collaboration

Given that improving collaboration was an important intermediate factor in the experiment investigated in this study, it also was one of the main themes. We found that important conditions and values for collaboration were stakeholders knowing each other in advance, having the same goal, understanding each other’s perspectives and trusting each other. These findings are in line with previous studies where the collaboration of mental health care and social security sectors on vocational rehabilitation were evaluated. These studies reported that maintained strong partnership and collaboration in a structural way were positive factors [14, 48].

Our study also found that even when there was a collaborative basis where people know and trust each other, barriers to collaboration like having different organizational structures and organization goals were still found. These findings are in line with work of Bergmark et al. 2019, who found that having conflicting goals was a barrier for achieving and consolidating collaboration when implementing supported employment. In our study, participants experienced that barriers could not easily be changed by themselves as they were based on legislation and historically grown organizational structures. Many other studies acknowledge that different organizational structures and working in different sectors can work as barriers for collaboration across sectors as it induces instability [14, 22, 25, 26]. Still, some participants found ways of working around these barriers and making the development of a new intervention fit into the existing structures. Steps that were important in achieving this were conducting a collaborative local needs assessment, identifying barriers and facilitators for the performance of a collaborative intervention, and setting up the collaborative intervention together. This is in line with implementation science literature that states that conducting a needs assessment and evaluating determinants for implementation are essential steps to develop an implementation plan [41, 43]. And those steps were found to be useful in the current study as well.

Above all, we found that improving client perspectives on societal participation and quality of life was mentioned as a personal driver by professionals from different sectors, organizational structures, and profession type. This motivation supported participants in working around collaboration barriers. Thus, our study suggests that focusing on a common goal based on personal motivators (working together in favour of the client and society outcomes) is a primary step for overcoming collaboration barriers in the development of an vocational rehabilitation intervention.

### Implementation versus financial and legislation aspects

In the present study, a shared savings strategy was used to overcome financial barriers for the implementation of a vocational rehabilitation intervention. Still several legislative and financial barriers came up during the experiment. Such as, not being allowed to purchase an intervention, not being allowed to reach or share financial and client data and information, and feeling hindered in collaborating because the uncertainty of a financial trade-off. These findings are in line with previous studies on the implementation of vocational rehabilitation interventions [19, 24, 26, 27] One particular study by Vukadin et al. (2018) about the implementation of IPS supported by improving stakeholder collaboration across the mental health care and social security sector also found financial barriers, even though a financial implementation strategy was used. The current study included similar stakeholders but the roadmap focused on the developmental process of setting up a collaborative intervention and then making collaborative funding agreements. But, similar doubts on conflicting goals (financial goals versus client goals) were mentioned by participants in both studies. While the study of Vukadin et al. found that the IPS professionals had no financial incentives, the current study found that at the level of management and directors the financial incentives were of a less importance when personal motivation driving force. Still, from both studies we could argue that focussing on financial aspects potentially distracts professionals from their personal drive to improve collaboration for better client outcomes.

Participants in our study specifically mentioned that financial gains were of less importance to them than client goals, but on the other hand funding facilitators were also mentioned. For example, the business case was seen as a supportive tool for decision making. Thus, participants acknowledged the function of financial insights in the process of agreement making, but financial incentives were not shown on an individual level.

When collaboration agreements were made during the experiment they were conducted on a small-scale pilot basis. Existing barriers were bypassed for these agreements. Financial agreements were not based on shared savings but were suited to the regional stakeholders wishes and possibilities. Moreover, funding agreements were not directly institutionalized via systems and established policy. Starting with a small-scale set up is in line with implementation science findings that starting with a small number is a good way to implement new intervention because it makes them more manageable [41, 49, 50]. Regarding to sustainable collaboration and funding there’s an interesting but complex balance between the success of a small-scale implementation and the needs for a broader and more sustainable imbedding of an intervention (scaling up) [49–51]. Qualitative monitoring the implementation of the developed interventions and moreover the effects of these agreements can add to sustainable imbedding.

### Contextual aspects and collaboration

The roadmap for sustainable funding based on a shared savings strategy used in the present study was performed in four different regions. We found that contextual factors, like regional structure, size of stakeholder and, role and history of collaboration differed by region and influenced the process of creating sustainable funding. Even so, themes that emerged were similar across all the regions. Our study showed important themes that can facilitate or hamper making financial and collaborative agreements to improve vocational rehabilitation interventions. The shared savings strategy used was largely based on similar initiatives in a different sector, namely the mainly therapeutic health care sector [33, 34, 52–54]. Contextual factors in those studies, such as the stakeholders involved, and the underlying goal of making shared savings agreements, differed from the contexts of the current study [34, 53, 55, 56]. Moreover, in the current study shared savings agreements were not achieved. However, similar findings on factors influencing collaboration (like trust and degree of collaboration) were described in the process of making funding agreements in the health care sector [56, 57]. Also the intrinsic motivation of helping client or patients are mentioned in playing a role in making agreements [57]. A large scoping review on bundle contracts (with or without shared savings agreements) in the health care sector even argued that implementation strategies should focus on long-term collaborative relationships [56]. These comparable findings on collaboration are interesting because contextual factors within the setting of this experiment differ considerably from the setting of the health care sector. Still, some main themes, especially on collaboration, arise in studies on financial implementation strategies aiming to improve clients or patients outcomes. Moreover, in both vocational rehabilitation and health care sectors, it is still under debate which factors are actually most important in strategies aimed at achieving improved quality of care (and shared savings) [57]. Qualitative studies on the implementation of funding agreements, like the current one, provides more insight into the process and contextual factors but also into underlying themes that can influence the effectiveness of these financial agreement programs.

### Strengths and limitations

Strength of this study is that, to our knowledge, it is the first study investigating a roadmap based on a shared savings strategy aiming to achieve sustainable funding for vocational rehabilitation services in the mental health care and social security sectors. This study showed in-depth insight into the process of making agreements on funding of vocational rehabilitation. While a lot of collaboration initiatives like this might exist in real life, only a fraction of these are being studied. We combined insights from multiple scientific fields and sectors to interpret the results and moreover raised insights that can be useful in those multiple fields.

Although the set-up of this study was not fully like participatory action research, some similar strengths and limitations of this approach can be seen during this study. The presence and participation of the researchers during the process in all four regions enabled us to obtain a lot of in-depth data, but the researchers could influence the process. Moreover, a large set of field notes were gathered from multiple and single stakeholder sessions in all four regions and interviews with participants in different professional roles (like project leaders, field professionals and steering board members) were conducted to enrich these field notes. A limitation is that a relatively low number of decision makers were interviewed, and further more in-depth knowledge of the political field of tension that decision makers occupy would add to the strength of this study. Only participants of sessions from the experiment were included, to study perspectives of the ones that were actually involved. A drawback of the experiment itself was, according to us, not including potential service users within the experimental process [58].

The COVID-19 pandemic was running during the term of the experiment. This was seen as both a strength and a limitation. The strength of this fact was that appointments and interviews were planned more easily because of the online options, enabling researchers to gain a lot of data in a short amount of time. However, the fact that almost all appointments took place remotely could also be seen as a limitation because participants could have experienced more distance and might have been less involved. Though no reluctance for participation was observed. The final limitation of this study was the fact that the intended endpoint of the roadmap (making financial agreements based on shared savings) was not reached in all four regions. Therefore, we cannot conclude whether the developed roadmap was suitable to make agreements based on investment and returning of the savings.

### Further research and practice

Practical implications—This qualitative study showed which themes were important in the process of improving sustainable funding between stakeholders across sectors, with the ultimate aim of improving vocational rehabilitation services for mental health care clients. Our study showed that factors, like personal motives on the client outcomes and aiming for the same goal in collaborations are found to be important facilitators in this process. These findings suggest that when aiming for collaborative agreements, it is important to start with personal drivers, and not with the financial incentive of overcoming unfair distribution. However, making a business case on collaboration agreements is still recommended, since according to participants, it can be helpful to support decision making. In addition, using the roadmap steps, supported by a process facilitator, was experienced to be helpful in developing a collaborative intervention. All of these lessons learned were integrated in an implementation guideline for the working field on how to make collaborative agreements, including a business case [59]. Stakeholders and policy makers who want to increase work participation of people with mental health problems can learn from this study on how to implement collaboration agreements on vocational rehabilitation.

Implications for further research—A lot of studies on the implementation of vocational rehabilitation have been published, primarily on supported employment [14, 19, 26, 28], but only one evaluated the actual implementation strategy used [24]. The current study adds on this and found factors that are important in collaboration on vocational rehabilitation. However, the study was performed over a limited period of time, namely during the period while regions were going through the roadmap. We suggest to follow stakeholders involved in vocational rehabilitation programs for a longer period of time, to gain more insight into the collaboration factors that remain important.

The ultimate goal of sustainable collaboration across the mental health care and social security sector is to improve long term perspectives on work participation and quality of life for people with mental health problems, thereby decreasing costs of social services and health care for this group. It is assumed that integrated vocational rehabilitation services are cost-effective. The business cases made for the interventions developed in this experiment also showed this, but to verify cost-effectiveness, outcomes of longitudinal data for the developed interventions should be evaluated.

## Conclusion

A new financial implementation strategy for improving collaboration on vocational rehabilitation services was introduced with the initiation of this experiment. This qualitative study showed that the roadmap based a on shared saving strategy helped stakeholders from mental health care and social security sectors to improve their collaboration, though the primary intended shared savings agreements were not achieved. We conclude that the roadmap supported stakeholders to establish more sustainable collaboration, even though no sustainable financial agreements were made yet. The driver for collaboration was found to be more on improving clients’ perspectives than on solving unfair financial distribution issues. It was shown that financial considerations were not the main driver for involved individuals, but personal motivation for helping clients was. Still, participants acknowledged the function of financial insights and resources to support decision making. This evaluation offered more insight into how collaboration on vocational rehabilitation can be improved.

### Supplementary Information

**Additional file 1.** Explanation of legislation and finance of vocational rehabilitation in the Netherlands. Additional file 1: Box 2 and Table 2: Explaining the Dutch context. Legislation and financial organization of mental health care, social security and vocational rehabilitation. Explanation of the Dutch context of legislation and financial organization of mental health care, social security and vocational rehabilitation. Including an overview of stakeholders, laws and tasks.

**Additional file 2.** Roadmap: Description of steps, activities per step and context per region. Detailed description of steps (including purpose and activities per step) and activities taken (including context) per region.

**Additional file 3.** Topic list interviews. Topic list used for interviews.

**Additional file 4.** Codebook MM. Codebook “Meedoen, Meedelen”: Overview of thematic order, used codes and sub codes. Translated from original codebook in Dutch. codebook on themes, codes and sub codes used to analyse the data.

## Data Availability

The datasets generated and/or analysed during the current study are not publicly available due privacy reasons of the participants but are available from the corresponding author on reasonable request.

## Abbreviations

IPS: Individual placement and support
SZW: Dutch Ministry of Social Affairs and Employment
SSI: The Dutch Social Security Institute
CIFR: The Consolidated Framework for Implementation Research
LDD: Labour-related developmental day care
JIT: Job-focused integrated treatment approach
w-CGT: Work-focused cognitive behavioural therapy
Wim: Work-focused interagency meetings
