# Supplementary material for: A roadmap for sustainable implementation of vocational rehabilitation for people with mental disorders and its outcomes: a qualitative evaluation
Source: Int J Ment Health Syst. 2024 Feb 10;18:7. doi: 10.1186/s13033-023-00620-8 (PMC10858636; doi:10.1186/s13033-023-00620-8)
Supplement: Supplementary file 1 — Additional file 1. Explanation of legislation and finance of vocational rehabilitation in the Netherlands. Additional file 1: Box 2 and Table 2: Explaining the Dutch context. Legislation and financial organization of mental health care, social security and vocational rehabilitation. Explanation of the Dutch context of legislation and financial organization of mental health care, social security and vocational rehabilitation. Including an overview of stakeholders, laws and tasks. [file 13033_2023_620_MOESM1_ESM.docx]

**Box 2: Explaining the Dutch context. Legislation and financial organization of mental health care and vocational rehabilitation.**

In the Netherlands, mental health care and social security services are organized in different systems (domains). Both systems follow multiple laws and financial flows. The application of vocational rehabilitation interventions for mental health care clients ask for an integration of services from both health care and social security , as clients mostly need support on both aspects. Stakeholders who are responsible for the application of vocational rehabilitation for mental health care clients work in favour of different laws. This box explains how laws, tasks and financial flows are regulated within the mental health care and social security systems.

**The application of vocational rehabilitation:**

The application of vocational rehabilitation interventions takes place on the border of the health care and social security systems and several stakeholders are involved. Mental health care providers are often responsible for the guidance and application of vocational rehabilitation interventions, in particular in supported employment. The Social Security Institute (SSI), depends on local policy, and job agencies or municipalities themselves can applicate vocational rehabilitation interventions.

From individuals perspectives, the application and regulation of a vocational rehabilitation intervention depends on two important factors:

1) which type of social benefit a client receives and from which organization (i.e., the SSI or municipality (and which municipality, as policies can differ per municipality) and;

2) whether or not a client receives a mental health care treatment.

**Health care system responsibilities and involvement in vocational rehabilitation:**

The (mental) health care system is regulated by the Health Insurance Act (Zorgverzekeringswet, ZVW) and the Long-term care Act (Wet langdurige zorg, Wlz) which are both national laws. Roughly divided, the Health Insurance Act is regulating ‘cure’ and the Long-term care act is regulating long-term care. Health care providers are responsible for the application of care from both laws. Mental health care providers are also responsible to guide people with mental health care to vocational rehabilitation, which is funded by health insurance act. Moreover, mental health care providers are also responsible for the application of vocational rehabilitation. But this is funded by the social security system.

**Health care system responsibilities and involvement in vocational rehabilitation:**

The social (security) services vary widely, from providing adult support services (see also box 1, main article), individual support, household help, vocational rehabilitation services to providing unemployment benefits and national (unemployment) insurances. Multiple benefit laws are in place ***, which are partly regulated locally by municipalities and partly country wide by the government. Moreover social support law are involved***, which are local laws provided by municipalities. The responsibility of the application of care and support from these acts depends on local policy. The other six country wide social benefits acts provided by the Dutch social security institute (SSI). See table 2 for an overview of laws, stakeholders and tasks.

***Table 2 overview of laws and tasks of the (mental) health care and social (security) system in the Netherlands***

**** The Unemployment Insurance Act (werkloosheidswet, WW), the Invalidity Insurance Act (wet op de arbeisongeschiktheidsverzekering, WAO) was replaced by the Work and Income according to Labour Capacity Act (wet werk en inkomen naar arbeidsvermogen, WIA), the Disablement Assistance Act for Handicapped Young Persons (Wet arbeidsongeschiktheidsvoorziening jonggehandicapten, Wajong), the Self-employed Persons Disablement Benefits Act (Wet arbeidsongeschiktheidsverzekering zelfstandigen (WAZ)) and finally the Sickness Benefits Act (Ziektewet, ZW). These are all country wide social benefits acts. The participation Act (participatiewet) is a local law.*

|  | **Health insurance act (Zvw)** | **Long-term care act (Wlz)** | **Social support act (Wmo)** | **Participation act (Participatiewet)** | **Social benefit acts ***** |
| --- | --- | --- | --- | --- | --- |
| **Level or regulation** | National law | National law | Local law | Local law | National law |
| **Aim of the law** | Arrange a social security system for health care | Arrange a social security system for health care | Arrange a social support system | Arrange a support system for income | Arrange a support system for income |
| **(financial) Arrangements of the law** | (obligated) Individual insurance added with social security | Social security of care | Social security of support | Social security of income | National insurance / Social security |
| **Regulating stakeholder *** | Health insurance companies | Care administration office | Municipalities | Municipalities | The Social Security Institute (SSI) |
| **Executive stakeholder **** | Health care providers | Health care providers | Social support providers or municipalities (depending on local policy) | Job agencies, mental health care providers, or municipalities (depending on local policy) | The SSI for providing benefits, job agencies, mental health care providers for application of vocational rehabilitation |
| **Tasks** (regarding mental health care, vocational rehabilitation and work) | Providing (mental) health care (cure) & guiding to vocational rehabilitation | Providing (mental) health care & guiding to vocational rehabilitation | Providing social support & guiding to vocational rehabilitation | Providing social benefits and provide guiding to vocational rehabilitation | Providing social benefits and provide guiding to vocational rehabilitation |

**Table 2: overview of laws, stakeholders and tasks.**

** regulating stakeholder means: stakeholder that decides whether individuals apply for the requested health care or security services.*

*** executive stakeholder means: stakeholder that delivers care or social support or benefit in case individual is eligible.*

**** The Unemployment Insurance Act (werkloosheidswet, WW), the Invalidity Insurance Act (wet op de arbeisongeschiktheidsverzekering, WAO) was? replaced by the Work and Income according to Labour Capacity Act (wet werk en inkomen naar arbeidsvermogen, WIA), the Disablement Assistance Act for Handicapped Young Persons (Wet arbeidsongeschiktheidsvoorziening jonggehandicapten, Wajong), the Self-employed Persons Disablement Benefits Act (Wet arbeidsongeschiktheidsverzekering zelfstandigen (WAZ)) and finally the Sickness Benefits Act (Ziektewet, ZW). These are all country wide social benefits acts. The participation Act (Participatiewet) is a local law.*
