# Supplementary material for: A roadmap for sustainable implementation of vocational rehabilitation for people with mental disorders and its outcomes: a qualitative evaluation
Source: Int J Ment Health Syst. 2024 Feb 10;18:7. doi: 10.1186/s13033-023-00620-8 (PMC10858636; doi:10.1186/s13033-023-00620-8)
Supplement: Supplementary file 2 — Additional file 2. Roadmap: Description of steps, activities per step and context per region. Detailed description of steps (including purpose and activities per step) and activities taken (including context) per region. [file 13033_2023_620_MOESM2_ESM.docx]

## Additional file 2 – Roadmap: Description of steps, activities per step and context per region.

| **Step** | **Purpose step** | **Activities step** | **Region 1** | **Region 2** | **Region 3** | **Region 4** |
| --- | --- | --- | --- | --- | --- | --- |
| 1. **Recruitment of stakeholders within regions** | Gather relevant representatives from every involved stakeholder (from the mental health care sector and social (security) services sector) to form a regional project group and steering group and sign declaration of intend. | - **Identify stakeholders** operating in the field of vocational rehabilitation and care/ social support of people with mental illness. - **Contact** representatives from every **stakeholder group**, at least one at the operational level, level of management and one director. - **Explain goal** of experiment to at least one (management or operational) representative of every stakeholder group (for example, policymakers, manager or job coaches), with this project group step 2-4 can be taken. - **Form a regional steering board** formed by directors from every participating stakeholder. - **Sign a declaration of intent** to continue with step 2-5, signed by the steering board. | **A project group** of relevant stakeholders (representatives from a mental health care provider, one municipality, two job reintegration agencies, the Social Security Institute (SSI) and the care administration office (‘zorgkantoor’)) already existed in this region, led by a (external appointed) regional project leader. So **stakeholders were already identified** and gathered easily for this experiment.  The project group existed of managers and policy makers from one municipality, two job(re-)integration agencies, a mental health care provider and a care administration office. The health care insurance company was not involved in this collaboration.  **The goal of the experiment** was made clear to all stakeholders by the project leader and were, supported by the regional project leader, linked to existing policy goals of the stakeholders in this region.  Stakeholders already had some agreements on collaboration or an intention to make them. Therefore, **an additional declaration of intent was not necessary** according to the stakeholders.  No specific **steering board** was formed, but directors were involved in decision making during the experiment, in case project group or leader felt this was necessary.  **Declaration of intent** was not signed by the involved directors. | The experiment was connected to an existing collaboration on mental health care and social care (a mental health care provider, a municipality and a welfare agency). Yet, work-participation was no key-theme of this collaboration and a social security professional was not involved in the team although represent in the steering board.  **Stakeholders were identified** easily and the external appointed regional project leader was already working for the existing collaboration. As a result, **a project group** was formed out of stakeholders engaged in the existing collaboration, complemented with job coaches from the municipality and the SSI^*^. The project group consisted of a mix of operational employees and managers per stakeholder, depending on the needs per step.  **Goal of the experiment goal** was made clear to the project group, by the project leader.  **A steering board** of relevant stakeholders already existed in this region, including a member from a health insurance company. The goal of the experiment was made clear to them by the project leader and was supported by the steering board (was found to be in line with existing goals).  **Declaration of intent** was signed by the steering board. | The experiment was placed as a follow-up of some previous collaborative initiatives. **Stakeholder representatives were gathered** easily based on these previous initiatives. A regional appointed project leader was active on this topic, but was replaced at the beginning of the experiment by a new project leader, on the pay roll of one of the stakeholders. Moreover, this second project leader had to quit during the experiment.  The **project group** consisted of a mix of operational employees and managers from a mental health care provider, a municipality, the SSI, a job reintegration agency and a mental client support organization.  a mental health care provider, a municipality, the SSI, a job reintegration agency and a mental health client support organization (‘clientondersteuner’)  Collaboration with the insurance company was mentioned difficult: they were hard to reach and showed no interest in the experiment.  Participation of some of the members ended during the experiment and replacement was not always arranged.  **Goal of the experiment** was clear for the project group.  **No steering board** was active in this region, though some directors and aldermen’s had personal interests in the target group (people with mental health problems) and were involved in the topic.  No steps were taken to form a steering group in this region and no **declaration of intent** was signed. | First, the experiment was seen as a follow-up from a pilot on supported employment which was running in this region. Yet, not all relevant stakeholders were involved in this pilot. Two mental health care providers were working together with a municipality and the SSI on this, though separately. so the project leader, on payroll of one of the pilot stakeholders, reached out to new stakeholders.  Primary focus of the collaboration was on the application of supported employment (IPS). Parallel some regularly policy meetings with regional municipalities were taking place.  Stakeholder representatives from SSI and a health care insurance company were invited to complement the project group. A SSI representatives participated incidentally and the health care company decided not to participate because this region had not their main focus and making this type of agreements did not match their policy goals.  Several meetings in multiple stakeholder compositions were arranged to explain the experiment and **identify the right stakeholder representatives.**  The final experiment project group consisted of the two mental health care providers and one municipality. Representatives from the SSI and a health care insurance company were invited to project group meetings; SSI representatives only participated incidentally and the health insurance company decided not to participate. Representatives from other municipalities were not invited for the experiment by the project leader.  **The goal of the experiment** was not always clear to members of the project group and needed to be explained multiple times.  Due to personal circumstances and interests, the project leader was replaced by a new person multiple times which took time.  Directors of the municipality and health care providers were having regularly meeting and supported the participation in the experiment**. Though, no regional steering board** was formed also no **declaration of intent** was signed. |
| **2a. Select a target group and develop a (collaborative) vocational rehabilitation intervention to stimulate work participation for this group.** | Select an suitable vocational rehabilitation intervention to improve work participation for a selected target group. | Schedule 3-5 interactive sessions with the regional project group to work on the next steps. Explore:   1. **current level of collaboration** between stakeholders. 2. **current offer** **of vocational rehabilitation interventions** of stakeholders offer and need for additional interventions. 3. **target group(s): unemployed people** with rehabilitation potential & needs, who could use extra attention in return to work process 4. **(financial) barriers currently** encountered in the collaboration of the application of vocational rehabilitation. 5. where **collaboration and application of vocational rehabilitation can be improved** to overcome these barriers.   Organize one-on-one sessions with stakeholders. Retrieve information about:  a. current work and caseload of operational employees.  b. (financial) barriers encountered in daily practice.  **Select a vocational rehabilitation intervention** for a selected target group aiming to overcome these (financial) barriers.  **Obtain information about financial aspects:**   - Work-related characteristics (like type of social benefit received, age) of a group of eligible clients. - Costs of individual stakeholders for the group of eligible clients in the current situation. (i.e. benefits costs, health care costs, social support costs, work-related costs, like wage costs). - Estimated costs every stakeholder makes for delivering the selected intervention. | **Current level of collaboration** of stakeholders was experienced good by project group members, which was also seen in the collaborative interventions that were developed recently.  **The current offer** was recently complemented with two new collaborative initiatives on vocational rehabilitation. These were the Labour-related developmental day care (LDD, intervention 1) and the job-focused integrated treatment approach (JIT, intervention 2) . These initiatives were initiated by members of the project group and were implemented on a pilot base.  *more information on developed interventions can be found in box Y.  Still, **improvements were seen for both pilots**: in the administration, the structural funding and support, the embedded application of the pilot into existing policy and organization, and in providing more insight in the actual effects for clients and costs.  Also, several barriers were mentioned like:  🡪 Clients were not always allowed to participate in the most available intervention (which was IPS) due to type of social benefits they receive. (=legislation barrier)  🡪 Pilot funding was not always arranged. (=financial barrier)  🡪 Interventions were running as part of a pilot so members had uncertainties about the continuation of this. (=financial barrier)  Information about **financial aspects** were partly obtained: work-related characteristics of participants of both type of pilots was available but mostly not structural gathered.  Data was kept separately by different stakeholders and it was not always allowed to share and compare this information, due to legislation.  Specific information on health care costs and other work-related costs was not available. The information needed for the business case was collected elsewhere, like from other research. | **Current level of collaboration** was experienced good by stakeholders, though work participation was no key theme in this collaboration. **The collaboration** was organized as add-on to an ongoing pilot where mental health care and social support professionals were operating in an integrated team.    **Current offer** was identified by the project group. Several interventions were available and several barriers on their implementation were mentioned:  🡪 Clients were not always allowed to participate in the most available intervention (which was IPS) due to their type of social benefits. (=legislation barrier)  🡪 the possibilities within and the duration of interventions were restricted. (=legislation barrier)  🡪 Geographical restrictions hindered clients from receiving the most appropriate support due to different policies. (=legislation barrier)  **Collaboration improvements** were identified by adding an supported employment (IPS^**^) specialist to the existing team, improving integral care of their caseload and broaden their vocational rehabilitation intervention options.  collaboration with SSI professionals in a more structural way and integrating (professionals on) vocational rehabilitation within the multidisciplinary teams.  A work-focussed interagency meeting (intervention 3) aiming to improve client perspectives on work possibilities was developed during the roadmap sessions.  Moreover, the project group was interest in implementing the job-focused integrated treatment approach (JIT) (intervention 2) from region 1.  *more information on developed interventions can be found in box Y  **Financial aspects:**  Due to a previous developed business case on a similar target group, a lot of work and health care related characteristics were easily available.  Specific information on work-related costs (like costs for benefits, health care costs and other work-related costs) were also available and obtained from the separate stakeholders. | Current level of **collaboration** of stakeholders was experienced good for professionals of the work floor and policy makers. But structural collaboration on directors and Alderman’s level was missing.  The **current offer** of vocational rehabilitation interventions was experienced sufficient, but the project group concluded that the organization of these intervention was not aligned due to legislation of financial restrictions and not in line with clients’ needs. (=legislation and financial barrier). For example: clients were not always allowed to participate in the most available intervention (which was IPS)  **Collaboration improvements** were identified by re-investing in a previous existing work-focussed interagency meeting (intervention 3). Where professional come together  for mental health care clients, aiming to improve clients’ perspectives on work possibilities.  (*more information on developed interventions can be found in box Y)  Information about **financial aspects** were partly obtained: work-related characteristics of the target were available, but data was kept separately by different stakeholders and it was not always allowed to share and compare, due to legislation and system features.  Specific information on health care costs and other work-related cost were not available. The information needed for the business case was collected elsewhere, like from other research. | Different types of **collaboration** were running, mostly based on a one-on-one relation. Regional municipalities came together regularly to exchange information on the topic of work-participation.  **The current offer** was identified by the project group. Available interventions were organized separately, depending on type of social benefits.  Several barriers were identified, like:  🡪 lack of funding for conducting vocational rehabilitation interventions. (=financial barrier)  🡪 Clients were not always allowed in the most available intervention (which was IPS) due to type of social benefits they receive. (=legislation barrier)  🡪 working with multiple regulations which are prone to changes (=legislation barrier).  Project group members had different interests and needs within the experiment, e.g., mental health care providers were interested in expanding the supported employment pilot, the municipality was interested in the outcomes of the research. Moreover, the municipality and SSI were also interested in exchanging and combining knowledge on vocational rehabilitation interventions. Getting consensus on the commons needs took several meetings.  The project group agreed to develop a business case on the running supported employment pilot.  Information about financial aspects was difficult to collect: work-related characteristics were not available or it was not allowed to share them due to privacy reasons.  Specific information on the costs of the supported employment pilot was available. |
| **2b. Develop business case of selected intervention** | To make a prognostic calculation about the costs and benefits of the (in step 2a developed) intervention for all involved stakeholders.  (These calculations of costs and benefits lay a foundation for making (financial) agreements on funding of the developed intervention (see step 4)). | Make a prognostic calculation (**business case**) based on obtained information about the financial and work-related outcomes.   - Determine the size of the selected target group and duration of intervention. - Calculate costs of the collaborative intervention. - Make a prognostic calculation on work participation of the selected target group, in case of care as usual and new situation. - Calculate costs and savings for both groups of clients for the current and new situation (include exact same costs as in current situation). - Make a prognostic calculation up to 4 years on outcome and success rates and per group of clients in case of new intervention and care as usual (control). - Divide costs and yields per stakeholder. | **Business cases** for both pilots were developed and turned out to be positive after one year of investment, calculated for a total of 4 years. Savings were primarily seen by the decreased use of social benefits and health care. | **Business cases** for the work-focussed interagency meeting and the JIT were developed. Both business cases turned out positive after one year of investment, calculated for a total of 4 years. Savings were primarily seen by the decreased use of social benefits and health care. | The **business case** on the intervention was developed and turned out positive after year one year of investment, calculated for a total of 4 years.  Savings were primarily seen by the decreased use of social benefits and health care. | The **business case** was partly developed, but due to lack of information and progress of the experiment, this exercise was discontinued and not resumed.  During this step a parallel countrywide initiative (on improving mental health care client’s perspectives on job participation) was introduced in this region, and connected to this experiment. Several regional meetings on this initiative were held and a new regional stakeholder was introduced. This led to a repetition of step 1-2b.  The project leader decided, in cooperation with national project group and regional stakeholder representatives, to prematurely **end the participation in the roadmap sessions as part of the experiment.** |
| **3. Make a detailed work plan about activities, quality indicators and costs** | To obtain a detailed plan where activities and costs are distributed over all participating stakeholders and quality indicators are set. | - Make a **workflow** ‘what and when by who’, **describe the distribution of activities** among the participating stakeholders, including activities on privacy, data exchange, use of systems etc. - Describe the **distribution of costs and savings** among included stakeholders - Set **quality indicators** to monitor the quality of the developed vocational rehabilitation intervention. (like throughout time per step or work participation per client). | Stakeholders already had operational agreements running in a pilot phase. Yet not every operational step was written out.  During the experiment **workflows** on both pilots were written out and a decision tree (to improve the allocation of clients to the most suited intervention) was developed. For example, inclusion criteria for clients and work participation were described in more detail.  Costs were already divided based on the previous agreements. The business case was added to future decision making on this.  Moreover, **quality indicators**, like throughout time, dropout rate and outflow to work, were set up more structurally. | A concept of **the workflow** and composition of the work-focussed interagency meetings were described: Like the composition and frequency of the interagency meetings and the client register procedure.  No specific **distribution of costs and savings** were made. The business case was discussed and added to future decision making on the financial aspects.  Aspects on privacy issues, the exchange of data and **quality indicators** were discussed but not described. | A concept of **the workflow** and a composition of the work-focussed interagency meetings were described.  Workflow was discussed several meetings and concretizing (distribution of) activities took some time. No specific distribution of costs were made. Aspects on privacy issues, the exchange of data and **quality indicators** were discussed but not described yet. | No specific intervention was developed and **no** **work plan** on activities, quality indicators and costs was made. |
| **4. Formalize work plan and costs and savings distribution in collaborative agreements** | To make agreements between all stakeholders about the implementation and funding of the developed intervention. | - Verify business case by the regional project group and steering group.   Let steering group making agreements on the work plan including at least:   - the selected intervention. - distribution of the activities of the application of the intervention. - the calculated distribution of costs and savings. - Formalize agreements for x amount of years in a contract Signed by the steering group members. | Both business cases were discussed and verified by several regional project group members and involved directors separately.  Directors involved in intervention 1 **decided** to continue the pilot of this intervention. A temporarily subsidy scheme was used for funding of the pilot and was embedded in local policy.  Intervention 2 was still running by the commitment of engaged professionals. But during the experiment involved managers and directors were working on arranging structural support which was not yet formalized. | As part of the roadmap sessions, a business case was discussed and verified in several regional project group meetings and in meetings with regional steering members. Business cases were presented to the steering board and steering board was overall positive of the collaboration agreements.  No **agreements** were made, because steering board felt not accountable to do so because the absence of the SSI board member.  Still intentions to make agreements were spoken out by the remaining members of the steering board:   - an intention to explore in which way the work-focussed interagency meeting can be steered more optimal (by a steering board with the necessary members) and - an intention to make agreements on the development of je JIT in in another way (attached to a nationwide deployment of this intervention). | The business case was discussed by the regional project group members in several meetings.  Involved directors and Alderman **agreed** to participate in a pilot version of the original developed intervention..  The municipality decided to support this by low-key pilot funding and organizational assistance. based on alderman’s decision. Business case was no part of this decision making, it was not yet verified by the project group and not presented to the Alderman.  Also agreements on extending the pilot were made, if pilot experience and outcomes turn out to be positive.  Informal low-key monitoring was part of the agreements and follow-up of this pilot was incorporated a in parallel countrywide initiative.  **Participation in the roadmap sessions as part of this experiment ended here.** | **No specific collaborative agreements were made** at this point, though regional stakeholders spoke out an ambition to improve their collaboration. |
| **5. Implement and monitor collaborative agreements** | Implement and monitor the developed intervention based on the agreements. | - Make an implementation plan on operationalisation the under step 3 developed work plan. - Create a **monitoring system** to monitor the under step 3 set quality indicators. - **Train** **staff** - **Communicate** about newly implemented intervention to relevant stakeholders and persons, like politics, professionals and client representatives. | When roadmap sessions ended, agreements on both pilots were still running. Initiatives for extending and consolidating were running.  **Staff was trained** on the application of both vocational rehabilitation interventions but no specific training plan was available. **Communication** on the interventions was running and in development.  **Monitoring** of both pilots was running and stakeholders were working on a more structural monitoring system. Quality indicators on the professional and client level were in development.  **Participation in the roadmap sessions as part of the experiment ended here.** | When roadmap sessions ended a supported employment (IPS^**)^ specialist was integrated to the neighbourhood team, but no implementation plan was made.  The first outlines of an implementation plan of the two other initiatives were in development.  For setting up the work-focussed interagency meeting a low-key implementation plan was in development.  The implementation of the work-related integral treatment approach was incorporated in a country-wide implementation plan of this intervention.  **Participation in the roadmap sessions as art of the experiment ended here.** | When roadmap sessions ended the first work-focussed interagency meeting was planned. | When roadmap sessions of this region ended no specific collaborative agreements were implemented and monitored. |

* SSI = Social Security Institute, ** IPS = Individual Placement and Support.
