# Supplementary material for: A roadmap for sustainable implementation of vocational rehabilitation for people with mental disorders and its outcomes: a qualitative evaluation
Source: Int J Ment Health Syst. 2024 Feb 10;18:7. doi: 10.1186/s13033-023-00620-8 (PMC10858636; doi:10.1186/s13033-023-00620-8)
Supplement: Supplementary file 3 — Additional file 3. Topic list interviews. Topic list used for interviews. [file 13033_2023_620_MOESM3_ESM.docx]

# Additional file 3 - Topic list interviews

**General information of the participant**

1. What is your current job function/ role?
2. What are your role and tasks and responsibilities in this function?
3. What are your experiences with supporting mental health care client in returning to work?
4. What do you think of work-possibilities for mental health care clients?
5. What is your role in the field of mental health care and/or social (security) services?
6. Do you work together with other stakeholders In your job?

**Role within the experiment**

1. What is your role within the experiment?
2. What are your tasks and responsibilities within this experiment?
3. How did you get involved within this experiment?
4. How is the experiment going so far?

***Perspective on experiment***

1. What is the goal of the experiment according to you?
2. What do you think of the ‘shared savings‘ principle?
3. Which risk and success factors do you see within the experiment?
4. Why would other stakeholder participate in this experiment?
5. Did COVID-19 impact the experiment?
6. Which steps of the experiment are useful for you?
7. Which steps of the experiment are less useful for you?

***Collaboration between stakeholder***

1. *With which stakeholders do you regularly collaborate in daily practice?*
2. *Do you collaborate with all stakeholder participation from the experiment?*
3. *How do you see the collaboration between the stakeholders in regular work?*
4. *Which barriers do you see in the collaboration with these stakeholders?*
5. *Which facilitators do you see in the collaboration with these stakeholders?*

***Barriers and facilitators on integrated care and vocational rehabilitation***

1. What are the most seen facilitators you encounter in your job?
2. What are the most seen barriers you encounter in your job?
3. What are barriers you of know of in supporting mental health care clients returning to work?
4. Do you think the experiment can overcome these barriers, and if so how?
