# Supplementary material for: A roadmap for sustainable implementation of vocational rehabilitation for people with mental disorders and its outcomes: a qualitative evaluation
Source: Int J Ment Health Syst. 2024 Feb 10;18:7. doi: 10.1186/s13033-023-00620-8 (PMC10858636; doi:10.1186/s13033-023-00620-8)
Supplement: Supplementary file 4 — Additional file 4. Codebook MM. Codebook “Meedoen, Meedelen”: Overview of thematic order, used codes and sub codes. Translated from original codebook in Dutch. codebook on themes, codes and sub codes used to analyse the data. [file 13033_2023_620_MOESM4_ESM.docx]

# Additional file 4 – Codebook “Meedoen, Meedelen”: Overview of thematic order, used codes and sub codes. Translated from original codebook in Dutch.

|  | | |
| --- | --- | --- |
| Theme | Code | Sub codes |
| Motives and interests | | |
| Personal motives and reasons to participate in the experiment | | |
|  | Interest of client | Helping out a client (+), improving (client) support (+), client first priority (+), investing in target group urgently needed (+). |
|  | Search for being valued | Feeling you are doing the right thing (+), don’t feel recognition for current work (+), interest in outcomes (effect)research (+). |
|  | Societal interest | Seeing the social value of experiment (+), motive to deliver social benefits (+). |
|  | Frustration | Frustration on how the current situation is (+), experiences of suboptimal support of clients (+), idealism (+), got to be another way (+), need to do it differently (+), wanting more freedom and flexibility in possibilities (+). |
|  | Finance not important | Money is not important (+), client above money (+), not participating in experiment for financial motives (+). |
| Personal motives and reasons for not participating in the experiment | | |
|  | Don’t have time | Too busy (-), don’t have time (-), to many things to do (-). |
|  | Not the right person | Not the right knowledge (-), not the right person (-), no authority for this (-), moving to another work position (-). |
|  | Already do things | Already working for mental health care and work participation (-), not added value for me (-). |
| Organizational reasons for participating in the experiment | | |
|  | Financial interests | Obtaining structural finance (+), compensate shortages (+),decreasing costs (+/-). |
|  | Quality of care motives | Improving organization services and support (+), deliver a higher quality of care/services (+), professional learning (+), smoothening work processes (+). |
|  | Expand tasks | Expand target group (+), helping more people (+), exploring new tasks (+),. |
|  | Decreasing number of clients /tasks | Decreasing social benefits clients (+), decreasing amount of support needed for clients(+). |
|  | Collaboration | Want to work together (+), want to do something for the target group together (+). |
| Organizational reasons for not participating in the experiment | | |
|  | Competing similar projects | Have similar running project (-), having pilot(s) (-), already have similar running research in this field (-). |
|  | No priority | No time (-), employees are busy (-), not of main interest (-), not responsible for achieving savings (-). |
|  | Limited profits | Too much work for little profits (-), little savings expected (-), not sure about the (social or financial) benefits (-). |
| Collaboration | | |
| Reason for wanting to collaborate (with other stakeholders) | | |
|  | Societal interests | Improving collaboration to foster society and gain societal value (+). |
|  | Knowledge | Exchange and improve knowledge (+), learning from each other (+), expanding network (+), making better choices (+). |
|  | More integral working | Reducing bureaucracy (+), diminish ‘from pillar to post’ (+), finding each other when needed (+), improve integral approach (+). smoothening work and collaboration processes (+), improving collaboration more important that finance (+). |
| Reasons for not wanting to collaborate (with other stakeholders) | | |
|  | Different goals | Only working in favour of own organizational goal (-), stakeholders having different goals (-), not having a focus on work participation or mental health (-), being in competition with stakeholders (-). |
|  | Other professional interests | Other stakeholder /professional would benefit from my effort, I do the work for somebody else. |
|  | Different view of target group | Different views on needs of people with mental health problem (e.g. needs on work or care). |
|  | No added value | Takes more time, don’t benefit from it myself, already collaborating, doing things double. |
| Values and characteristics of collaboration | | |
|  | Knowing each other | Know each other (+), having a collaboration network(+), contact each other faster(+), know how to find each other(+),collaborated before (+/-), new participant don’t know people (-), never contacted before(-). |
|  | Trust | Having trust in each other (+), collaboration feels good (+), know wat to expect from each other (+), don’t trust each other (-), can’t rely on each other (-). |
|  | Aiming for the same goal | Aiming for the same goal (+), having conflicting goals (-). |
|  | History of collaboration | Collaborated before (+), good previous experience(+), bad previous experience (-). |
|  | Time and investments | wanting to invest (+), takes time and devotion (+/-), need a driving force (+/-), need finance (+/-), |
|  | Mutual interests | We have a common interest (+), different interests needs to be clear (+/-), other organization has no stake in (-), our organization has no stake in (-). |
|  | Important persons in work group | Important link (+), access to stakeholders / right persons (+), being a driving force (+), Project leader role (+/-), push agenda setting (+/-). |
|  | Continuity and structure of work group | agenda of meetings (+/-), replacement of participant (-), project leaders stops (-),, rescheduled/cancelled meetings (-), resetting goal (-) and explaining goal multiple times (-). |
| Collaboration structure and organization | | |
|  | Geographical organisation | Stakeholder working in the same geographic field (+), stakeholder working in partly overlapping geographic fields (+/-), stakeholder not working in the same geographic field (-). |
|  | Changing collaboration structures | Change in reimbursement agreements (+/-), change in collaboration agreements (+/-), new stakeholder involved (+/-), stakeholder left collaboration (-). |
|  | Level of collaboration | Collaboration of operational level and policy/management (+/-), collaboration of policy/management and politics/directors (+/-), collaboration of operational level and politics/directors (+/-). |
| Politics, finance and legislation | | |
| Politics | | |
|  | Political trade-off between stakeholders | Dual interests (+), political sentiments (+/-), opposite interests(-), want something in return (-). |
|  | Political support experiment | Aldermen’s or directors interest (+/-), needs of aldermen or director (+/-), asking for political support (+/-), political responsibility (+/-), level of authority, who makes decisions (+/-). |
|  | Timing | Timing of involving political leaders or directors (+/-), timing of decision making (+/-), political hassle (-). |
|  | Decision making | Business case is supportive (+), need a substantive base (+/-), evidence based for outcomes (on finance or client perspectives) (+/-). |
| Finance | | |
|  | Business case | Great interest in (+), data difficult to reach (-), data not available (-), complicated (-), abstract (-), no clients story (-). |
|  | Financial incentives | What are the savings (+/-), where are the saving going to (+/-), money doesn’t play a role (+/-), organization is financially driven (+/-). |
|  | Financial barriers | Funding criteria (-), not allowed to finance (-), lack of financial recourses (-), financial aspects hinders collaboration (-), bureaucratic process to obtain funding (-). |
|  | Financial transactions /trade-off | financial commitment (+/-), unfair distribution of funding (-), who will fund (-). |
| Legislation | | |
|  | Legislation barriers | Privacy (-), no funding allowed (-), legislation prohibits (-), system restrictions (-), rules not in line with original intention (-). |
|  | Changing legislation | New national scheme with overlapping purpose (+/-), new law changes collaboration (-), legislation will not change (-). |
|  | Dependency on legislation | We need to justify (+/-),our goal is set by legislation (+/-), difficult to break through (-), cannot change (-). |
| Complexity of a new experiment | | |
| Goal and concept of the experiment | | |
|  | Goal unclear of experiment | client goals (+), collaboration goal (+/-), discussion on goals (+/-), assignment unclear (-), financial goal (+/-). |
|  | Complexity assignment | Determining a target group (+/-), define the scope (+/-),process of concretising (+/-), restricting possibilities (-), expectations unclear (-), process of returning of the savings (-). |
| Organizational aspects | | |
|  | Participants of roadmap sessions | Participants missing (-), participants don’t show up (-), need another participant (-), authority of participant (+/-), right participants (+). |
|  | Connection to other initiatives | good alignment (+), connection to existing programs (+/-) , multiple initiatives (+ /-), bad alignment (-) , overlapping (+/-). |
|  | Preconditions experiment | Funding of the experiment (+/-), information provision (+/-), justifying to authority (-), terms not clear (-), obligatory activities (-), communication(+/-). |
|  | Support experiment | Supporting the sessions (+), guiding to next step(+), concretising of roadmap steps (+), calculating the business case(+/-), Explaining the experiment (+/-), |
|  | Size experiment | Duration of the experiment (+/-), number of clients needed (-), number of involved stakeholders (-), size of research (-). |
